# Genetic Characterization and Statistical Interpretation of 16 STR Markers in South-West Bulgaria: Implications for Forensic Identification and Kinship Analysis

**DOI:** 10.3390/genes17040493

**Published:** 2026-04-21

**Authors:** Vera Djeliova, Bogdan Mirchev, Ekaterina Angelova, Milka Mileva, Dimo Krastev, Atanas Hristov, Yanko Kolev, Aleksandar Apostolov

**Affiliations:** 1 Department of the Molecular Biology of the Cell Cycle, Institute of Molecular Biology “Roumen Tsanev”, Bulgarian Academy of Sciences, Akad. G. Bonchev Str., Bl. 21, 1113 Sofia, Bulgaria; vera@bio21.bas.bg; 2 DNA Laboratory, Department of Forensic Medicine and Deontology, University Hospital “Alexandrovska”–1, “St. Georg Sofiiski” Blvd., 1431 Sofia, Bulgaria; bogdanmirtschew@gmail.com (B.M.); ekvaan@mail.bg (E.A.); 3 Department of Forensic Medicine, University Hospital “Burgas”, 8000 Burgas, Bulgaria; 4 Department of Virology, Institute of Microbiology, Bulgarian Academy of Sciences, 26 Acad. Georgi Bonchev Str., 1113 Sofia, Bulgaria; 5 College of Medicine “Yordanka Filaretova”, Medical University of Sofia, 1606 Sofia, Bulgaria; dimo_krustev@mail.bg; 6 Department of Forensic Medicine and Deontology, Faculty of Medicine, Medical University of Sofia, 2 Zdrave St., 1431 Sofia, Bulgaria; ahristov@medfac.mu-sofia.bg; 7 Department of Forensic Medicine, Intern of Medical Imaging, MBAL “Gabrovo” District Hospital, 5300 Gabrovo, Bulgaria; drforensic@gmail.com

**Keywords:** DNA analysis, STR, Bulgarian population data, STR population database, NGM Detect^TM^ PCR Amplification Kit

## Abstract

**Background/Objectives:** The widespread adoption of short tandem repeat (STR) marker technology in genetic analysis has led to the collection of substantial STR data from diverse populations. Allele-frequency data provide robust forensic utility and support accurate likelihood ratio calculations, highlighting the importance of regional databases. **Methods**: The presented study aimed to determine the allelic frequencies and statistical parameters for 16 autosomal genetic STR markers included in the NGM Detect^TM^ PCR Amplification Kit in a population sample of 220 unrelated individuals from the South-West region of the Republic of Bulgaria. **Results**: We found that the most polymorphic and informative marker for the Bulgarian population in the southwestern region is SE33, with the next most informative markers being D1S1656, D12S391, D18S51, and FGA. In contrast, D22S1045, D16S539, and D2S441 showed comparatively lower genetic variability and informativeness. At the same time, no deviations from the Hardy–Weinberg equilibrium were observed for the 16 loci studied. **Conclusions**: This work not only enriches knowledge of the genetic diversity of the Bulgarian population but also provides the Bulgarian and international justice systems with an objective, scientifically sound basis for expert decision-making.

## 1. Introduction

**Figure 1:**
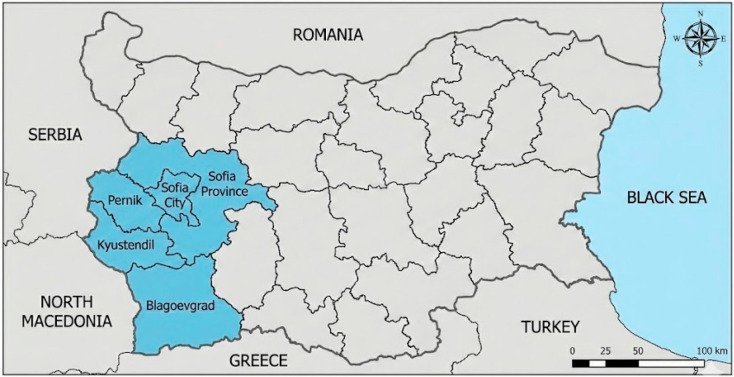
Map of Bulgaria showing the South-West region [1].

Bulgaria is located in the eastern part of the Balkan Peninsula. The southwestern region of Bulgaria occupies a strategic geographical position, bordering Serbia to the west, North Macedonia to the southwest, and Greece to the south. It includes the areas around the cities of Pernik, Sofia City and Sofia province, Kyustendil, and Blagoevgrad (Figure 1) [1]. The Balkans were the main bridge over which the first farmers from Anatolia settled in Europe around 8000 years ago [2]. The southwestern region (along the Struma River Valley) was part of the “Southern Arc,” a corridor connecting Asia and Europe, which appears to have served as a key migration route for human movement, including gene flow, from the Neolithic to the migrations of the 20th century [3].

Human identity testing using short STR loci has become a cornerstone of modern forensic genetics. Accurate statistical interpretation of DNA evidence requires population-specific allele-frequency databases that reflect regional genetic structures. The likelihood ratio (LR), widely used in forensic casework, compares the probability of observing a DNA profile if it originates from the suspect versus a random individual from the population. It is highly dependent on allele frequencies, emphasizing the importance of localized population data. Probabilities are calculated for each STR locus under the population hypothesis and combined across loci to obtain the overall LR, providing a measure of the evidential weight of the profile [4,5].

Due to its geographical location at the crossroads of Europe and Asia, Bulgaria has experienced complex demographic processes that have contributed to genetic heterogeneity within its population [6,7,8,9]. Although several studies have reported genetic data for the Bulgarian population [7,8,9], regional genetic analyses remain essential, not only for advancing theoretical knowledge, but also for supporting practical applications, particularly in forensic and judicial investigations involving the identification of unknown individuals [5].

Extended STR multiplex systems significantly improve the efficiency of modern forensic practice by increasing discriminatory power and exclusion capacity. The NGM Detect^TM^ PCR Amplification Kit represents a significant technological advance, including 16 autosomal STR loci, including SE33 [10]. SE33 is known for its exceptional polymorphism and utility in the analysis of complex family relationships, including paternity testing. In this context, the availability of a reliable database on the frequency of the specified variants in the STR system within a given population is of utmost importance.

STR genotyping of non-coding polymorphic loci provides the basis for individual genetic profiling. Allelic variation arises primarily through replication slippage and DNA repair mechanisms, contributing to genetic diversity across populations [11,12]. Although extensive STR data exist for European and global populations, representative regional studies for Bulgaria remain quite limited [8]. To obtain reliable genetic parameter estimates, forensic laboratories rely on population databases containing various genetic markers, such as autosomal STRs [13,14,15]. These databases must accurately reflect the genetic structure of individuals in the region where the tests are conducted. Furthermore, it is crucial that they are regularly updated to maintain the quality and accuracy of forensic analyses. In this context, establishing accurate allele-frequency databases is essential for forensic analysis, kinship analysis, and judicial expertise when there are individuals from this region among the suspects [13,14,15].

This study aimed to determine allelic frequencies and statistical parameters for 16 autosomal STR loci in 220 unrelated individuals from the South-West region of the Republic of Bulgaria and to establish reliable allele frequency and forensic statistical parameters for regional forensic applications. The following forensic statistical parameters were calculated: expected heterozygosity (Hexp), observed heterozygosity (Hobs), power of discrimination (PD), polymorphism information content (PIC), probability of match (PM), exclusion power (PE), typical paternity index (TPI) and Hardy–Weinberg equilibrium (pHW) (*p*-value). Observed heterozygosity (Ho) reflects the proportion of heterozygous individuals at a locus, and expected heterozygosity (He) represents the probability of heterozygosity under random mating. The polymorphism information content (PIC) quantifies how informative a marker is by measuring its level of polymorphism. Among core forensic statistics, probability of match (PM) estimates how likely it is that two random individuals share the same genotype at a locus, while power of discrimination (PD) indicates the likelihood that two unrelated individuals can be distinguished. For kinship analysis, the power of exclusion (PE) reflects the proportion of unrelated individuals that can be excluded in paternity testing, and the typical paternity index (TPI) evaluates how many times more probable it is that the tested father is the biological parent compared to a random individual. The Hardy–Weinberg (HW) equilibrium detects whether allele frequencies in the analyzed population deviate from expectation, a key preliminary step in forensic data quality assessment [13,14,16].

## 2. Materials and Methods

### 2.1. Population DNA Sampling

Biological samples (buccal swabs) were obtained from 220 healthy, unrelated individuals originating from the South-West region of Bulgaria, specifically covering the administrative districts of Blagoevgrad, Kyustendil, Sofia, and Pernik. To ensure the genetic representativeness of the local population, only volunteers whose families had resided in the region for at least three generations were included in this study. This research was carried out in the DNA laboratory at the Department of Forensic Medicine and Deontology at the University Hospital “Aleksandrovska” in Sofia. To enter the generated DNA profiles into an information array, the “Familia’s” software [17] was used. Declarations of informed consent were signed by examined living persons for the provision of obtained samples. In the informed consent declaration, only the volunteers’ regional origins were disclosed, in compliance with the Republic of Bulgaria’s and the EU’s legislative requirements for data protection. Samples were anonymized before laboratory processing to ensure privacy and data protection.

### 2.2. DNA Extraction and Quantification

DNA was isolated using Chelex 100 extraction [18] from collected buccal swabs. DNA quantification was performed with a QuantifilerTM Trio DNA Quantification Kit—Thermo Fisher Scientific 168 Third Avenue, Waltham, MA 02451, USA on the Real-Time PCR system 7500 (Life Technologies LTD, 7 Kingsland Grange, Woolston Warrington WA1 4SR UK) for HID analyses, and quantitative evaluation of the DNA present in the samples by HID Real-Time PCR Analysis Software v1.2.

### 2.3. DNA Amplification

Multiplex PCR amplification was performed with 0.5 ng of genomic DNA for 16 somatic loci and the Amelogenin sex-determining system included in the NGM Detect^TM^ PCR Amplification Kit (Life Technologies LTD, 7 Kingsland Grange, Woolston Warrington WA1 4SR UK) in 25 µL reactions, according to the manufacturer’s instructions [10]. The NGM Detect^TM^ PCR Amplification Kit is an extended European Standard Set of twelve loci (European Standard Set, ESS)—FGA, TH01, vWA, D3S1358, D8S1179, D18S51, D21S11, D12S391, D1S1656, D2S441, D10S1248 and D22S1045—plus four additional loci that are not in the European Standard Set, but present in the AmpFℓSTR^TM^ SGM Plus^TM^ DNA Amplification PCR Kit: SE33, D16S539, D2S1338, and D19S433. It should be noted that the SE33 marker is one of the most polymorphic loci introduced in these kits for human identification. Also, the NGM Detect^TM^ PCR Amplification Kit includes two internal quality control markers (IQCS and IQCL)—indicators for degradation of the DNA in a sample or for the presence of DNA inhibitiors, one insertion/deletion polymorphic marker on the Y chromosome (Y indel) and Amelogenin (sex-determining marker). The amplification of STR markers was performed using the Simpli Amp^TM^ Thermal Cycler (Life Technologies).

### 2.4. Fragment DNA Analysis

Fragment DNA analysis was performed on a Genetic Analyzer model 3500 Series Genetic Analyzer for Human Identification (Life Technologies) by eight capillary electrophoreses (with the 3500 POP-4^TM^ Polymer) with fragment laser detection and computer analysis using Gene MapperTM v1.2 Full Software (Life Technologies) for HID analysis [19]. The sizes of the observed peaks (above 50 RFU), quality, presence or absence of interferences and possible null alleles were assessed. The control and standardization of analyses were carried out by positive control—DNA Control 007; negative control; Matrix Standard DS-33 3500 Series (6-FAM^TM^, VIC^TM^, NED^TM^, PET^TM^, and LIZ^TM^ dyes); internal standard—GeneScan^TM^ 600 LIZ ^TM^ Size Standard v2.0; internal quality control markers—IQCS and IQCL; and an allelic witness (NGM Detect^TM^ Allelic Ladder) for the relevant STR markers, validated and embedded in the NGM Detect^TM^ Kit (Applied Biosystems, Waltham, MA, USA).

### 2.5. Statistical Analysis

Generated DNA profiles were entered into an information array [17] and analyzed. Statistical analyses of generated DNA profiles were performed with the following software products: FORSTAT—Forensic Statistics analysis toolbox (https://fdl-uwc.shinyapps.io/forstat, accessed on 2 April 2026) [20] and STRAF Software Version STRAF 2.2.2. https://straf-p7bdrhm3xq-ew.a.run.app (accessed on 2 April 2026) [21]. In order to characterize the 16 autosomal STR markers, the following forensic statistical parameters were calculated: Hexp, Hobs, PD, PIC, PM, PE, TPI and pHW (*p*-value). The Hardy–Weinberg equilibrium (HWE) test was performed using an exact test with 1000 permutations to assess whether the observed genotype frequencies at each locus deviated from those expected under random mating. A *p*-value greater than 0.05 indicated no significant deviation from the equilibrium, confirming that the loci conformed to HWE expectations [13,14,21].

## 3. Results

### 3.1. Allelic Frequencies and Statistical Parameters of 16 STR Loci

**Figure 2:**
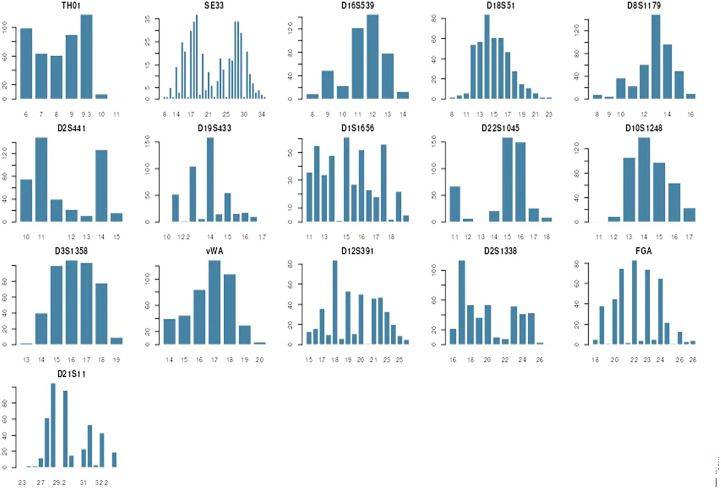
Graphical representation of counted allele data in population group of 220 individuals (16-marker NGM Detect^TM^ system) [20].

The allele frequencies of 16 autosomal genetic markers (STR) for 220 unrelated individuals from the South-West region of the Republic of Bulgaria were estimated using statistical programs FORSTAT—Forensic Statistics analysis toolbox [20] and STRAF Software Version STRAF 2.2.2 [21]. Graphical representations of counted allele data in the population group of 220 individuals are presented in Figure 2. The allelic frequencies of 16 STR loci are presented in Table 1. The statistical parameters of 16 autosomal genetic markers (STR) for the Bulgarian population (*n* = 500) were calculated using the statistical program STRAF Software Version 1.0.5. [21]. Calculated statistical parameters are summarized in Table 2.

### 3.2. Allelic Diversity and Polymorphism

A total of 152 alleles were identified in the 16 loci studied. The number of alleles varied significantly, which is an indicator of the rich genetic heritage of the region. The SE33 locus showed the highest number of alleles (35). The D10S1248, D16S539 and TH01 loci showed the lowest number of alleles (7) but nevertheless retained high informativeness. It was found that markers SE33, FGA, D21S11, D12S391 and D18S51 showed greater variability.

### 3.3. Forensic Efficiency

Statistical parameters confirmed that this set of markers is an extremely powerful tool for forensic practice. We found that the most polymorphic and informative marker for the Bulgarian population in the southwestern region is SE33, with the next most informative markers being D1S1656, D12S391, D18S51, and FGA. The lowest levels of PIC, PD, and PE, and the highest values of PM and homozygosity, were observed for three markers: D22S1045, D16S539, and D2S441. Allele frequencies of all 16 studied autosomal STR markers had PIC values higher than 0.5, which meets the condition of being sufficiently informative for the studied population [22]. The SE33 value (0.944) is among the highest reported for Balkan populations. These data show that for the needs of pathoanatomy and cadaver identification, these markers provide exceptionally high levels of statistical certainty. The average TPI for SE33 was 13.750, which allows for categorical conclusions even in the absence of one parent or when working with degraded biological samples. All 16 loci are in the Hardy–Weinberg equilibrium. This is a critical result, which proves that: (i) the sample is representative of the population in Southwestern Bulgaria; and (ii) these data can be used directly to calculate the Likelihood Ratio (LR) in forensic examinations without the risk of statistical errors arising from isolated genetic groups [4,13,14,22].

## 4. Discussion

Historically, Bulgaria’s location in the eastern Balkans has made it a crossroad of civilizations for millennia [1,2,3]. This geographic positioning represents a key factor contributing to the high genetic diversity observed in both previous studies and in the present analysis of 220 unrelated individuals from the South-West region of Bulgaria [7,8,9,23]. Based on the analysis of 16 STR markers contained in the NGM Detect^TM^ PCR Amplification Kit [10], it was found that the most polymorphic and informative locus for human identification of the Bulgarian population is SE33. This marker has the highest PIC, PD, PE, PI, and the lowest frequency of MP and homozygosity. However, it is considered a challenging locus due to the presence of numerous isoalleles that cannot be resolved by standard capillary electrophoresis and may require sequencing for accurate genotyping [5]. The next most informative markers are D12S391, D1S1656, D18S5.1, and FGA. These markers have the highest PIC, PD, PE, and PI, and the lowest MP and homozygosity. Conversely, D22S1045, D16S539 and D2S441 showed lower polymorphism and forensic efficiency.

Observed heterozygosity ranged from 0.732 to 0.941 and closely matched expected heterozygosity values. In addition, all loci conformed to the Hardy–Weinberg equilibrium (Table 2). The lack of significant deviation from the Hardy–Weinberg equilibrium validates the genetic representativeness of our South-West Bulgarian sample set [13,14,22].

According to Butler J.M., the effectiveness of a marker for human identification depends on three causally related factors—allele diversity, heterozygosity, and resulting discriminatory power—that align with the patterns observed in the present study [24].

A recent DNA population study of the Bulgarian population was conducted, including calculations of allele frequencies and statistical parameters at identified genetic loci [8]. It was found that the Bulgarian population has formed an exceptionally rich gene pool, most likely due to the country’s geographical location and the historical movement of people of different nationalities through the territory of the Republic of Bulgaria over the centuries.

Conducting such DNA population studies contributes significantly to the collection and construction of the world’s library of population-level DNA characteristics. Such a library is of great value for judicial and criminological investigations of serious cases, such as the identification of unidentified corpses or the resolution of cases of disputed origin. In this sense, the results obtained in this study should be disseminated to support comparative analyses of DNA profiles of individuals presumed to belong to the Bulgarian population. In fact, conducting regional population studies allows comparing these results with data on populations from neighboring and European countries in order to assess potential genetic proximity.

These generated allele-frequency data provide high identification value in forensic casework, including mixed DNA interpretation, analysis of unidentified remains, and kinship calculations. Establishing regional databases is essential for accurate statistical evaluation and reliable likelihood ratio calculations [4,13,14,15].

The presence of rare alleles and high heterozygosity observed in the population of Southwestern Bulgaria is undoubtedly a reflection of the region’s complex demographic history. Serving as a major trans-Balkan crossroads, the area has experienced multiple genetic influxes, including Neolithic migrations and Roman-period mobility, as well as significant waves of refugees from the Aegean and Vardar regions in the early 20th century. This historical ‘admixture’ resulted in a robust genetic reservoir, as evidenced by our STR analysis. The results from this study support the need to continue regional STR studies to strengthen national and international forensic genetic databases and improve the statistical interpretation of DNA evidence.

Studies on different Balkan countries (Bulgaria, Bosnia, Serbia, and Romania) and subpopulations, e.g., Karakachans and Roma, illustrate significant genetic diversity within the region. The results of Benvisto et al. (2018) even show that this diversity can be so pronounced as to warrant treating some regional groups as separate entities in forensic databases [24].

Our findings on allelic distributions in the population of Southwestern Bulgaria provide empirical support for regional patterns of diversity previously identified in the Balkans [25]. Compared with larger datasets from neighboring countries such as Serbia and Romania, our results are consistent with the hypothesis of Benvisto et al., which suggests that some Balkan subpopulations show a level of genetic differentiation that cannot be ignored in high-precision forensic work [24]. The high heterozygosity and polymorphic content observed with the NGM Detect^TM^ kit in our sample (*n* = 220) indicate that although the southwestern group shares a common Bulgarian genetic core, there are subtle but statistically significant variations. This reinforces the need for localized reference databases. As Benvisto argues, failing to account for such regional stratification can lead to an overestimation of the rarity of specific genotypes, potentially affecting likelihood ratio (LR) calculations in criminal investigations. Therefore, our data serve as a critical refinement to Bulgaria’s national DNA database, providing more reliable and ethically justified forensic evidence [24].

The genetic landscape of the Balkans is further complicated by the presence of diverse ethnic and social isolates, such as the Sarakatsani (Karakachani) and the Roma population. The Sarakatsani, former nomadic shepherds practicing transhumant pastoralism, display genetic signatures shaped by long-term endogamy and isolation. Similarly, the Roma population exhibits a unique genetic profile, resulting from founder effects and subsequent drift. As highlighted by Benvisto and co-workers, the existence of such stratified groups within the same geographical boundaries necessitates the development of precise, region-specific forensic databases to ensure the reliability of DNA-based evidence [26].

Rare alleles and high heterozygosity in South-western Bulgaria underscore a complex demographic past. Historically a trans-Balkan crossroads, the region has integrated various genetic lineages, including Neolithic settlers, Roman-era populations, and early 20th-century refugees from the Aegean and Vardar regions. As evidenced by our STR analysis, this prolonged genetic exchange has fostered a highly diverse genetic landscape.

The results of this study support the need to continue regional STR studies to strengthen national and international forensic genetic databases and improve the statistical interpretation of DNA evidence.

## 5. Conclusions

This study provides a comprehensive forensic and population genetic characterization of the South-West Bulgarian population using the 16-locus NGM Detect^TM^ system. Our analysis of 220 unrelated individuals confirms that this region exhibits exceptionally high discriminatory power and genetic polymorphism, making it a robust reference for forensic identification and kinship testing.

From the 16 genetic markers examined in the individuals from the South-West region of the Republic of Bulgaria, the most polymorphic and informative forensic identification locus was SE33. The most informative markers were D1S1656, D12S391, D18S51, and FGA. The lowest levels of PIC, PD, PE, and PI, and the highest values of MP and homozygosity were observed for three markers: D22S1045, D16S539, and D2S441.

Beyond their technical utility, these data underscore South-West Bulgaria’s status as a genetic melting pot within the Balkan Peninsula. The high allelic richness and heterozygosity observed are consistent with the region’s role as a historical and geographic crossroads. The Struma River valley, acting as a primary migration corridor, has facilitated millennia of gene flow between the Mediterranean basin and the Balkan interior, integrating Paleo-Balkan, Roman, Slavic, and more recent migratory lineages.

In conclusion, the established allele-frequency database not only enhances the precision of forensic expertise in Bulgaria but also contributes to a broader understanding of human genetic diversity in Southeastern Europe.

## Figures and Tables

**Table 1:** Allelic frequencies of 16 STR loci for the Bulgarian population in the South-West region (*n* = 220).

Allele	D10S1248	D12S391	D16S539	D18S51	D19S433	D1S1656	D21S11	D22S1045	D2S1338	D2S441	D3S1358	D8S1179	FGA	SE33	TH01	vWA
6															0.225	
7															0.145	
8			0.020	0.005								0.018		0.002	0.139	
9			0.111									0.011			0.205	
9.3															0.268	
10			0.052	0.009	0.002					0.170		0.084			0.016	
11	0.002		0.277	0.014		0.082		0.152		0.339		0.052			0.002	
11.2														0.002		
11.3										0.091						
12	0.020		0.330	0.123	0.118	0.125		0.016		0.050		0.139		0.011		
12.2					0.005											
13	0.241		0.180	0.130	0.239	0.077		0.002		0.025	0.005	0.339		0.002		
13.2					0.014											
14	0.316		0.030	0.191	0.361	0.109		0.048		0.289	0.091	0.220		0.032		0.091
14.2					0.034									0.007		
14.3						0.002										
15	0.223	0.030		0.139	0.125	0.139		0.361		0.036	0.227	0.114		0.057		0.102
15.2					0.036											
15.3						0.061										
16	0.145	0.036		0.139	0.041	0.118		0.341	0.050		0.243	0.023		0.048		0.191
16.2					0.023											
16.3						0.052								0.002		
17	0.052	0.082		0.107	0.002	0.041		0.059	0.259		0.236			0.068		0.293
17.3		0.023				0.127										
18		0.191		0.064		0.005		0.020	0.123		0.177		0.011	0.077		0.245
18.3		0.014				0.050										
19		0.120		0.034					0.084		0.020		0.086	0.084		0.068
19.2													0.002	0.005		
19.3		0.025				0.011										
20		0.114		0.025					0.123				0.102	0.045		0.009
20.2														0.009		
20.3		0.002														
21		0.105		0.014					0.023				0.170	0.027		
21.2													0.005	0.014		
22		0.107		0.005					0.018				0.189	0.002		
22.2													0.009	0.018		
23		0.075		0.005			0.002		0.118				0.168			
23.2													0.011	0.041		
24		0.045							0.095				0.148			
24.2							0.005							0.030		
25		0.020							0.098				0.050	0.002		
25.1													0.002			
25.2														0.041		
26		0.011					0.005		0.007				0.030			
26.2														0.039		
27							0.027		0.002				0.007			
27.2														0.077		
28							0.141						0.009			
28.2														0.077		
29							0.239									
29.2							0.002							0.066		
30							0.218							0.002		
30.2							0.034							0.048		
30.3							0.002									
31							0.052									
31.2							0.120							0.025		
32							0.007									
32.2							0.098							0.016		
33							0.002							0.007		
33.2							0.043							0.009		
34														0.005		
34.2							0.002							0.002		

**Table 2:** Statistical parameters of 16 STR loci for the Bulgarian population in the South-West region (*n* = 220).

Locus	N	Nall	GD (Hexp)	PIC	PM	PD	Hobs	PE	TPI	*p*_Value
D10S1248	440	7	0.770	0.731	0.095	0.905	0.795	0.591	2.444	0.791
D12S391	440	16	0.897	0.886	0.022	0.978	0.877	0.749	4.074	0.746
D16S539	440	7	0.768	0.730	0.096	0.904	0.809	0.616	2.619	0.434
D18S51	440	15	0.878	0.863	0.031	0.969	0.886	0.768	4.400	0.837
D19S433	440	12	0.780	0.749	0.083	0.917	0.773	0.549	2.200	0.067
D1S1656	440	14	0.902	0.891	0.024	0.976	0.927	0.851	6.875	0.275
D21S11	440	17	0.847	0.827	0.045	0.955	0.864	0.722	3.667	0.256
D22S1045	440	8	0.725	0.678	0.125	0.875	0.745	0.502	1.964	0.937
D2S1338	440	12	0.862	0.845	0.037	0.963	0.841	0.677	3.143	0.240
D2S441	440	7	0.762	0.724	0.092	0.908	0.705	0.435	1.692	0.222
D3S1358	440	7	0.795	0.761	0.084	0.916	0.850	0.695	3.333	0.149
D8S1179	440	9	0.796	0.767	0.074	0.926	0.800	0.599	2.500	0.293
FGA	440	16	0.865	0.848	0.037	0.963	0.882	0.758	4.231	0.228
SE33	440	35	0.949	0.944	0.010	0.990	0.964	0.926	13.750	0.326
TH01	440	7	0.797	0.763	0.075	0.925	0.782	0.566	2.292	0.882
vWA	440	7	0.796	0.764	0.081	0.919	0.836	0.668	3.056	0.459

Abbreviations: N: number of alleles for 220 individuals in locus; Nall: number of observed alleles in locus; GD (Hexp): expected heterozygosity; PIC: polymorphism information content; PM: probability of match; PD: power of discrimination; Hobs: observed heterozygosity; PE: power of exclusion; TPI: typical paternity index; *p*-Value—pHW of Hardy–Weinberg equilibrium exact test (number of permutations: 1000).

## Data Availability

The raw data supporting the conclusions of this article will be made available by the authors on request.
